# Supplementary material for: An adaptive peptide-binding site in ubiquitin receptor hRpn13 revealed by structural studies
Source: Nat Commun. 2025 Jul 1;16:5669. doi: 10.1038/s41467-025-60843-w (PMC12218921; doi:10.1038/s41467-025-60843-w)
Supplement: Supplementary file 1 — Supplementary Information [file 41467_2025_60843_MOESM1_ESM.docx]

**
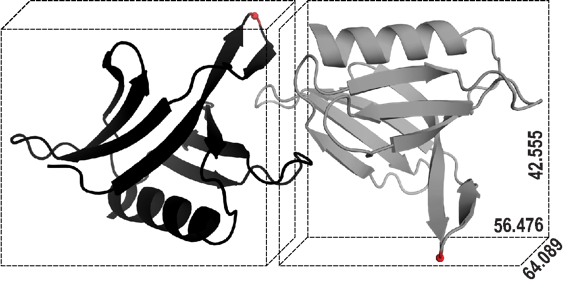
a b**

**c**


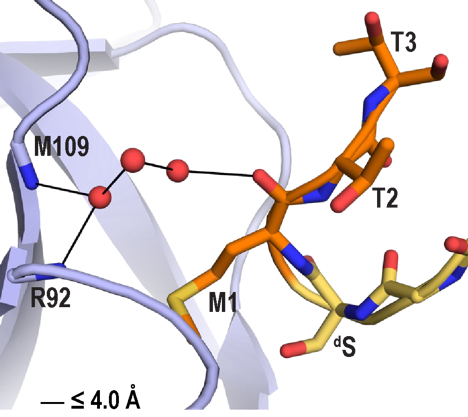


**d e**

**** **f g**

**Supplementary Figure 1: Features of the hRpn13 Pru:ENT structure. a)** Arrangement of protomers in a previously solved hRpn13 Pru apo structure (PDB 5IRS) with the unit cell indicated by a black box and unit cell dimensions included in Å. Red spheres indicate the Cα atom of G35. **b)** Superimposition of the hRpn13 Pru displayed as a ribbon diagram from a previous study (PDB 5IRS, black) and our study (purple) with the additional electron density displayed as grey mesh. The β6/β7 loop differs between the two structures and is indicated. All secondary structural elements are labeled. **c)** An expanded view of the overlaid structures in panel **(b)** to highlight the variance of the β6/β7 loop region spanning P89 – G91. The electron density map and stick display of the heavy atoms is included for Q87 – R92 (grey mesh) for the ENT-bound structure. Carbon, oxygen and nitrogen are colored purple, red and indigo, respectively. **d)** Ribbon diagram of Rpn2 (green) bound hRpn13 Pru (purple, PDB 6CO4) with a stick view of sidechains V38, C88, R92, W108 and E111 highlighting the proteasomal binding site where hRpn13 is anchored to the proteasome through Rpn2. **e**, **f)** Electrostatic interactions (black lines) between ENT and Pru mediated by bound water molecules. Coloring follows figure 1d for ENT, with the oxygen atom of water molecules displayed as a red sphere. **g)** Structure of ENT (native sequence in orange, non-native sequence in yellow) bound at the interface of three Pru molecules (labeled Pru-A, Pru-B and Pru-C) in the crystal lattice. A hydrogen bond between ^a^G and V69 is displayed. The ENT molecules from the Pru-B and Pru-C asymmetric units are hidden.

**
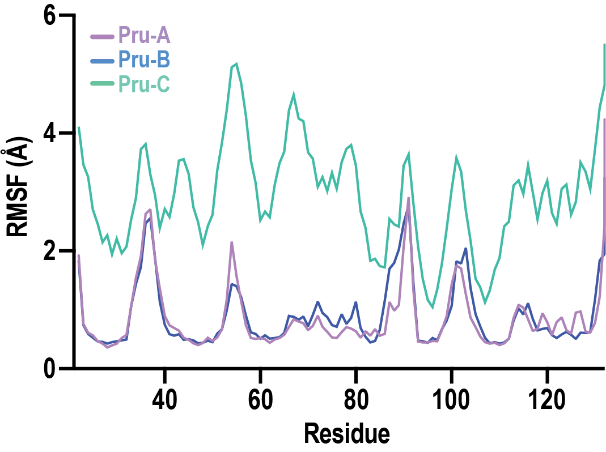

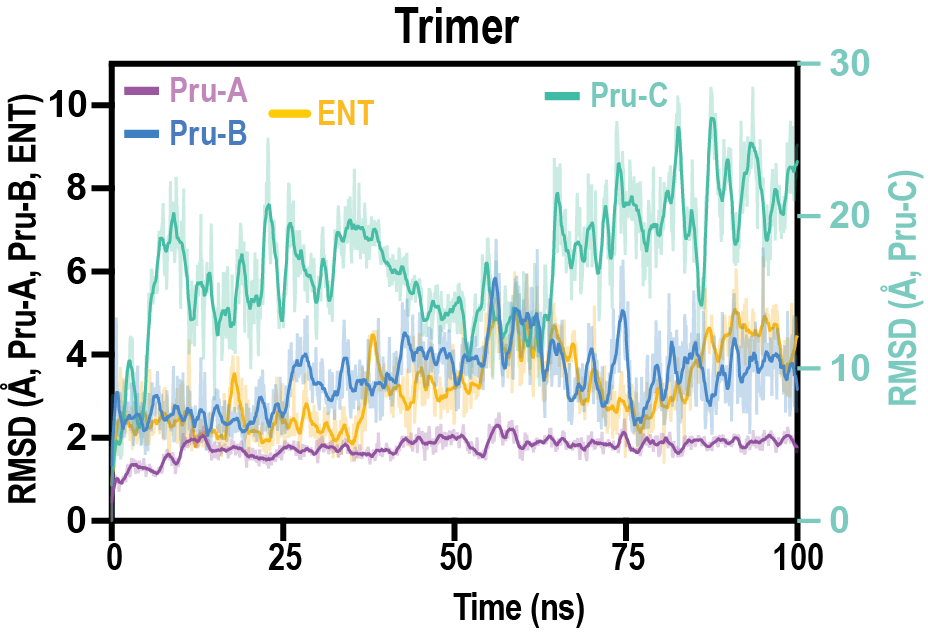
a b**

**Supplementary Figure 2: MD simulations on the hRpn13 Pru trimeric complex with ENT yields large RMSD and RMSF values for Pru-C. a)** Averaged C_α_ RMSD values over 100 ns of MD simulations for Pru-A (purple, left axis), Pru-B (blue, left axis), Pru-C (blue-green, right axis) and ENT (orange, left axis) within the trimeric Pru:ENT complex relative to the first frame of Pru-A. Plotted values are averaged value over 2 ns (dark line) with individual data points displayed. **b)** Overlay of averaged C_α_ RMSF values for the Pru-A (purple), Pru-B (blue), or Pru-C (blue-green) residues over the 100 ns of MD simulations for the trimeric Pru:ENT complex relative to the starting frames of each Pru molecule. Source data for **a** and **b** are provided in the Source Data file.

**a**

**b**

**Supplementary Figure 3: ENT remains embedded between Pru-A and Pru-B throughout three extended MD runs of 200 ns. a)** In the trimeric Pru:ENT complex, distances are plotted for three extended MD runs of 200 ns (n=1001) for hRpn13 Pru-A P40 Cβ to M1 Cα and K42 Nζ to carbonyl of T2; Pru-B K99 Nζ or D78 Cγ to carbonyl of ^a^G; and Pru-C N68 Oδ1 to ^a^G amide nitrogen. Median values are represented by dashed lines and the first/third quartile are represented by dotted lines. **b)** Ribbon representation of the last frame following 200 ns of extended MD simulations for the trimeric Pru:ENT complex displaying Pru-A, Pru-B, Pru-C, and ENT from the final frame in purple, blue, green, and orange/yellow, respectively. The first frame (T_0 ns_) or x-ray structure (for run 1) is overlayed and displayed in grey. Source data for **a** is provided in the Source Data file.

**
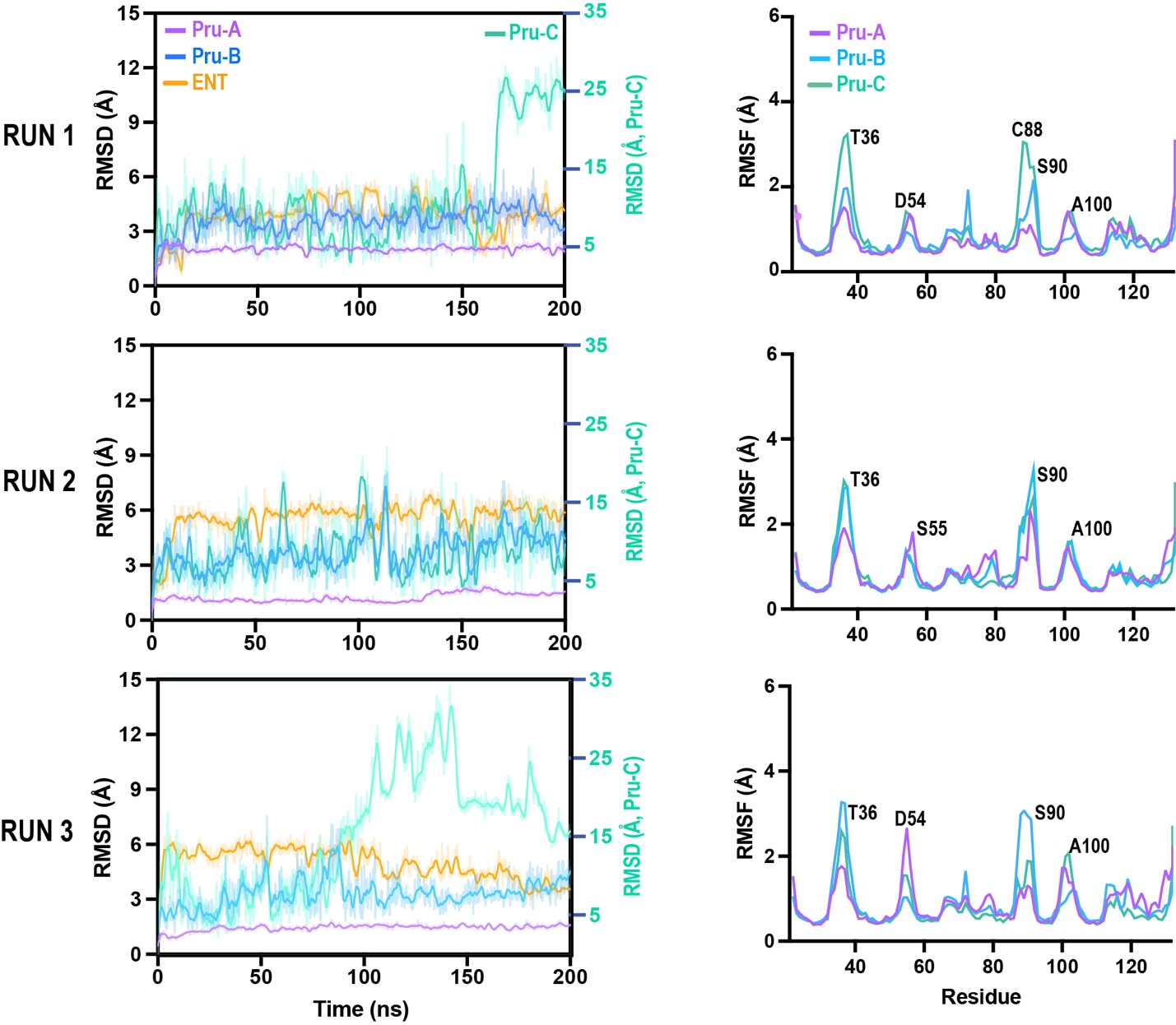
a b**

**Supplementary Figure 4: RMSD and RMSF values for 200 ns of extended MD simulations for the trimeric Pru:ENT complex. a)** C_α_ RMSD values over time for three independent replicates of MD simulations for the trimeric Pru:ENT complex plotting the averaged value over 2 ns (dark line) with individual data points for Pru-A (purple, left axis), Pru-B (blue, left axis), Pru-C (blue-green, right axis) and ENT (orange, left axis) relative to the first frame of Pru-A following the initial 100 ns of MD. **b)** Overlay of averaged C_α_ RMSF values for the individual Pru-A (purple), Pru-B (blue), or Pru-C (blue-green) residues from the trimeric Pru:ENT complex over 200 ns of MD simulations relative to the first frame of each respective Pru molecule. Source data for **a** and **b** are provided in the Source Data file.

**
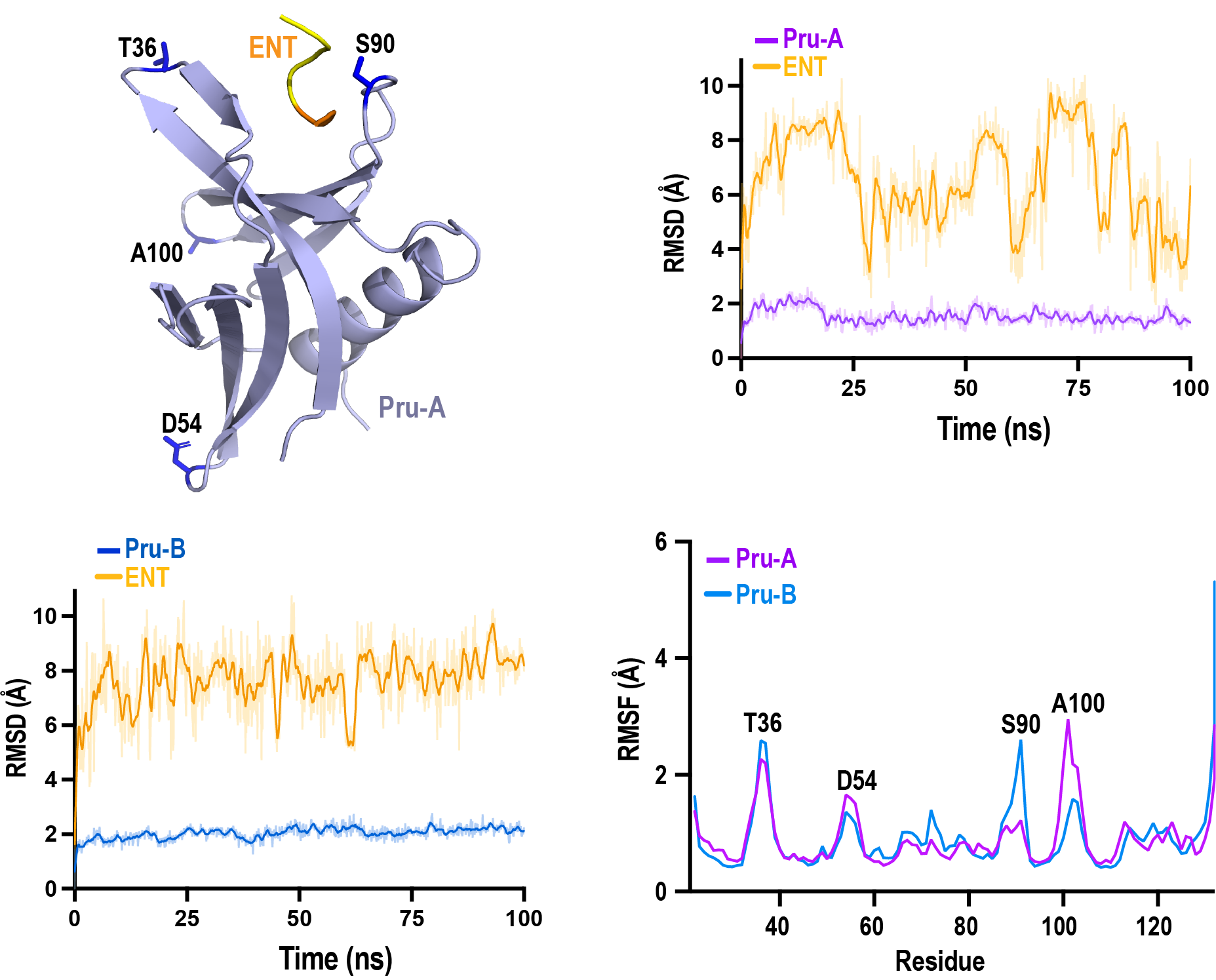
a b**

**c d**

**Supplementary Figure 5: RMSD and RMSF values for 100 ns of MD simulations for Pru-A or Pru-B complexes with ENT. a)** Ribbon representation of hRpn13 Pru-A (purple) complexed with ENT (orange and yellow) to highlight the positions of T36, D54, S90, and A100 at the center of loop structures. The sidechain heavy atoms of these amino acids are displayed as sticks. **b, c)** C_α_ RMSD values over time for 100 ns of MD simulations for (b) Pru-A or (c) Pru-B complexed with ENT plotting averaged value over 2 ns (dark line) with individual data points for Pru (purple or blue) and ENT (orange). **d)** Overlay of averaged C_α_ RMSF values for Pru-A (purple) or Pru-B (blue) from Pru:ENT complexes over 100 ns of MD simulations. Source data for **b, c** and **d** are provided in the Source Data file.

**a**

**b**

**Supplementary Figure 6: Averaged atom pair distances throughout extended MD runs of 200 ns. a, b)** Distances are plotted for three extended MD runs of 200 ns (n=1001) for the (a) hRpn13 Pru-A or (b) hRpn13 Pru-B complex with ENT. Distances are between Pru-A M1 Cγ, S, or Cβ and P40 Cβ, S90 Oγ, or W108 Cε3 respectively and P40 Cβ and ^d^S Cβ. Distances for Rpn13 Pru-B are between F76 Cε2 to M1 Cε, D78 Cγ to ^a^G or ^c^G backbone nitrogen, D79 Cγ to ^d^S backbone nitrogen, and K99 sidechain Nζ to carbonyl of ^a^G or ^b^P. Median values are represented by dashed lines and the first/third quartile are represented by dotted lines. Source data for **a** and **b** are provided in the Source Data file.

**
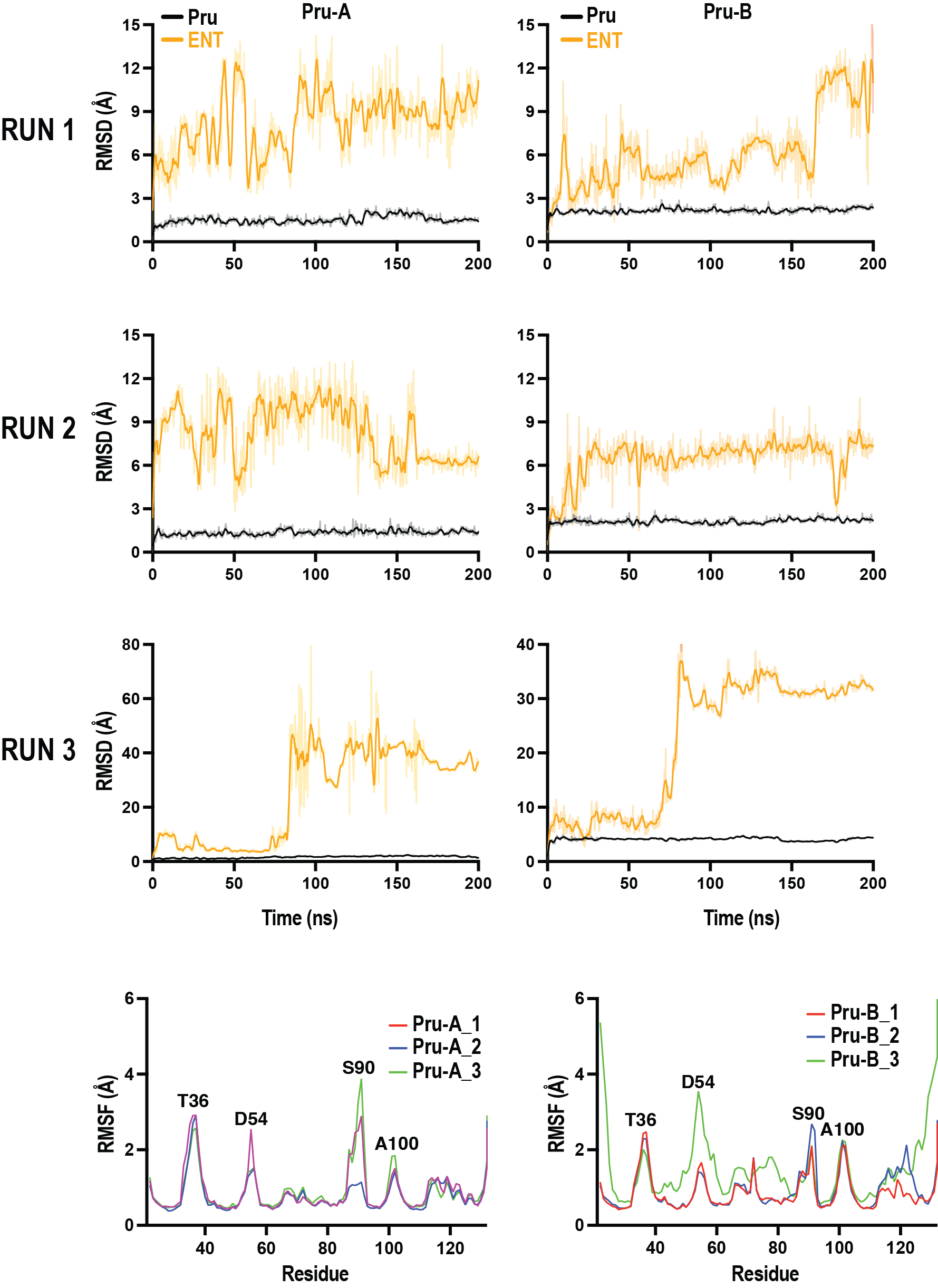
a**

**b**

**Supplementary Figure 7: RMSD and RMSF values for 200 ns of extended simulations for Pru:ENT complexes. a)** C_α_ RMSD values over time for three independent replicates of MD simulations for Pru-A:ENT (left) and Pru-B:ENT (right) complexes plotting the averaged value over 2 ns (dark line) with individual data points for Pru (black) and ENT (orange). **b)** Overlay of averaged C_α_ RMSF values for the individual Pru-A (left) or Pru-B (right) residues from Pru:ENT complexes over 200 ns of extended MD simulations. Source data for **a** and **b** are provided in the Source Data file.

**
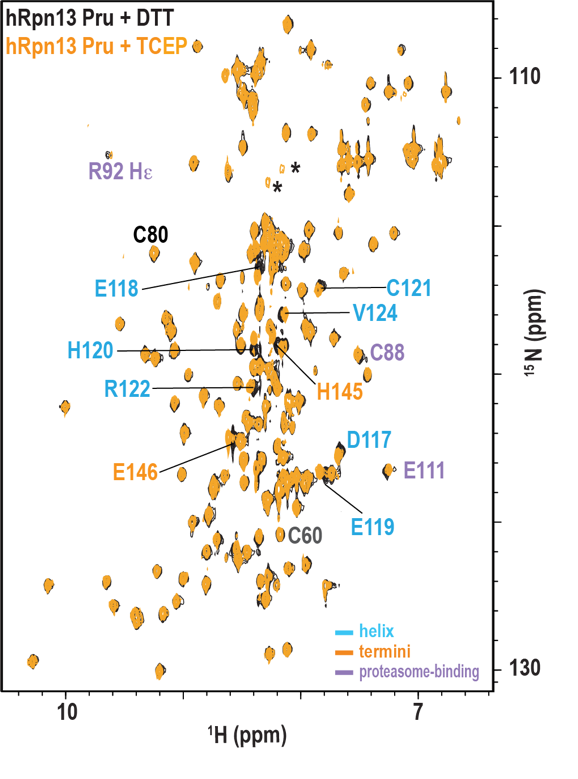
a b**


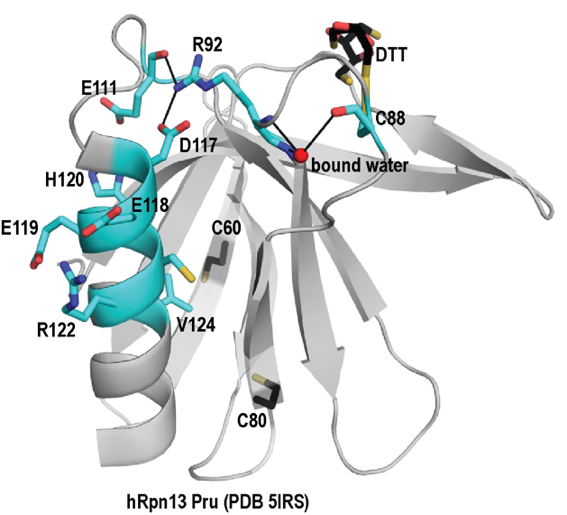


**c**

**Supplementary Figure 8: Comparison of 2D ^1^H-^15^N HSQC spectra of hRpn13 (1-150) with either DTT or TCEP present in the buffer. a)** Overlaid ^1^H-^15^N HSQC spectra of 0.1 mM ^15^N-hRpn13 Pru dissolved in NMR buffer with 1 mM DTT present (black) or following exchange into NMR buffer with 1 mM TCEP and dilution to 0.04 mM ^15^N-labeled hRpn13 Pru (orange). Signals from cysteine and those that shift are labeled. ppm, part per million. **b)** Enlarged views of regions from the ^1^H-^15^N HSQC spectra of panel **a** highlighting signals from the helix (blue font), hRpn2-binding surface (purple font), or C-terminal end (orange) that are perturbed by replacement of DTT with TCEP. The spectra were acquired at 850 MHz and 25°C. **c**) Ribbon diagram of DTT-bound hRpn13 Pru (grey, PDB 5IRS) highlighting residues sensitive to DTT substitution for TCEP in blue and showing in stick view their sidechain atoms with nitrogen, oxygen, and sulfur in indigo, red, and yellow respectively. C60 and C80, which do not shift, are displayed with stick rendering showing carbon and sulfur in black and yellow respectively. Oxygen from a bound water molecule is displayed as a red sphere and labeled, DTT is displayed with carbon, oxygen, and sulfur in black, red, and yellow respectively.

**
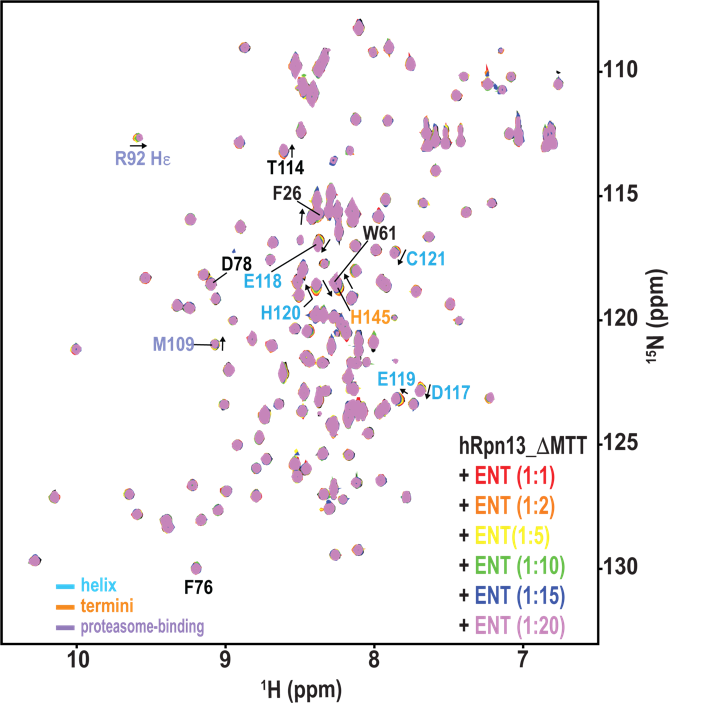

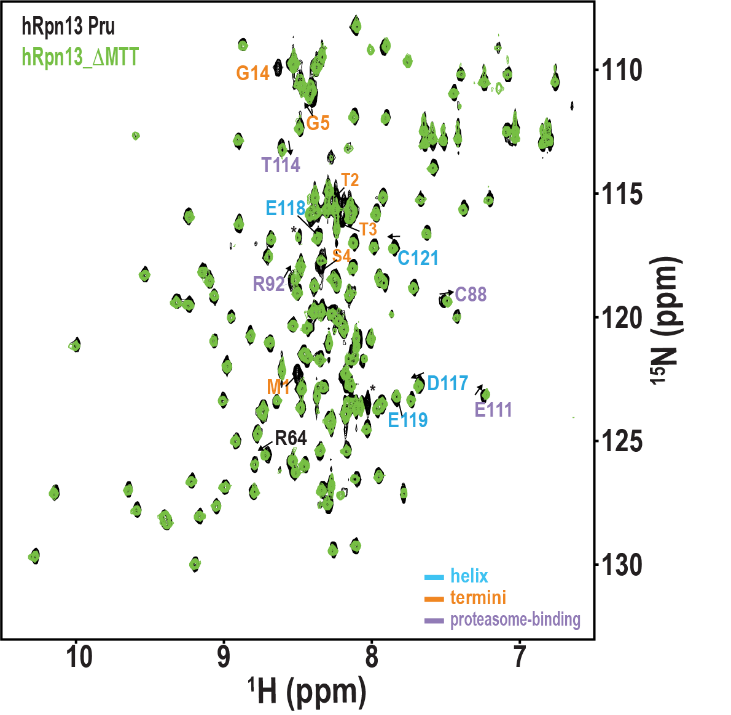
a**   **b**

**
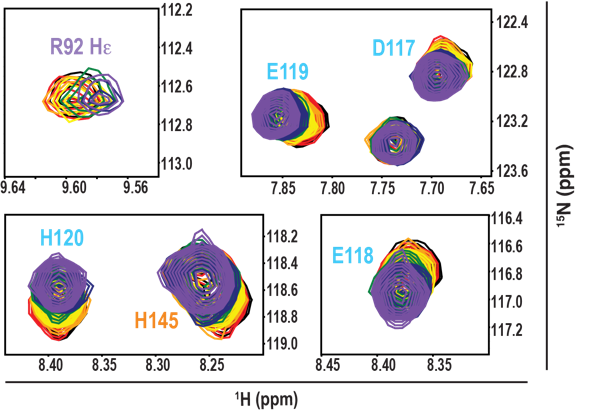
**

**c**

**Supplementary Figure 9: 2D NMR experiments indicate ENT binding to hRpn13 Pru. a)** ^1^H-^15^N HSQC spectrum of 0.1 mM ^15^N-labeled hRpn13 (1-150, black) or hRpn13_ΔMTT (green). Select residues are labeled with amino acids in the Pru helix, proteasome binding region, and termini in blue, purple, and orange, respectively. Arrows indicate direction of shifting. **b, c)** ^1^H-^15^N HSQC spectra of 0.1 mM of ^15^N-labeled hRpn13_ΔMTT alone (black) or with ENT present at equimolar (red), 2-fold (orange), 5-fold (yellow), 10-fold (green), 15-fold (blue), or 20-fold (purple) molar excess. Magnified views of regions with signals that shift are displayed in panel **c** and denoted in panel **b**. All spectra were acquired at 600 MHz and 25°C in 20 mM sodium phosphate, pH 6.5, 100 mM NaCl, 1 mM TCEP, 10% D_2_O. ppm, part per million.

**
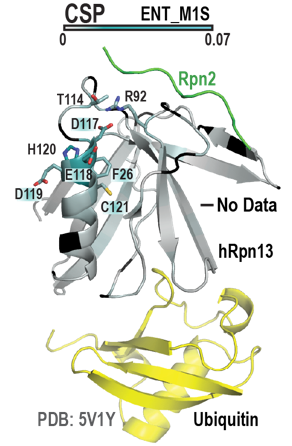

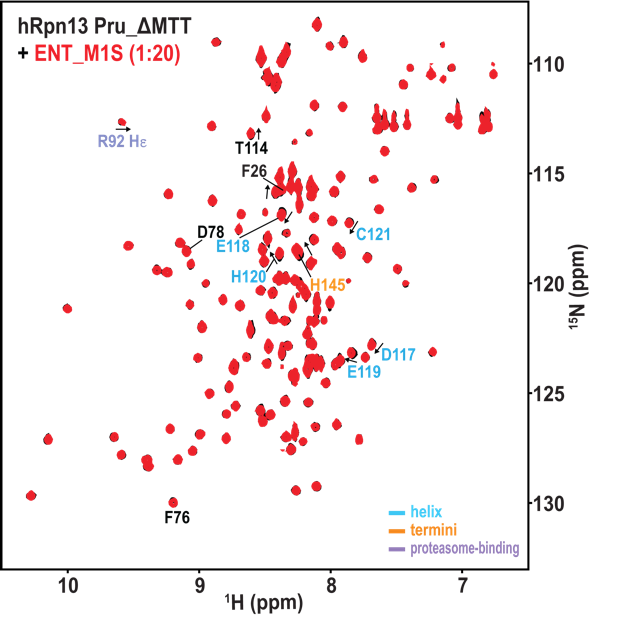

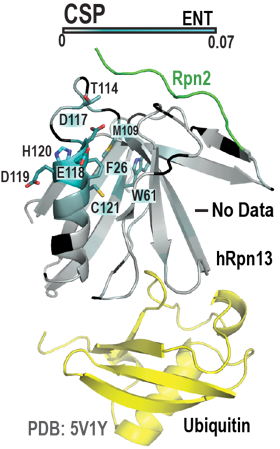
a**  **d e**

**
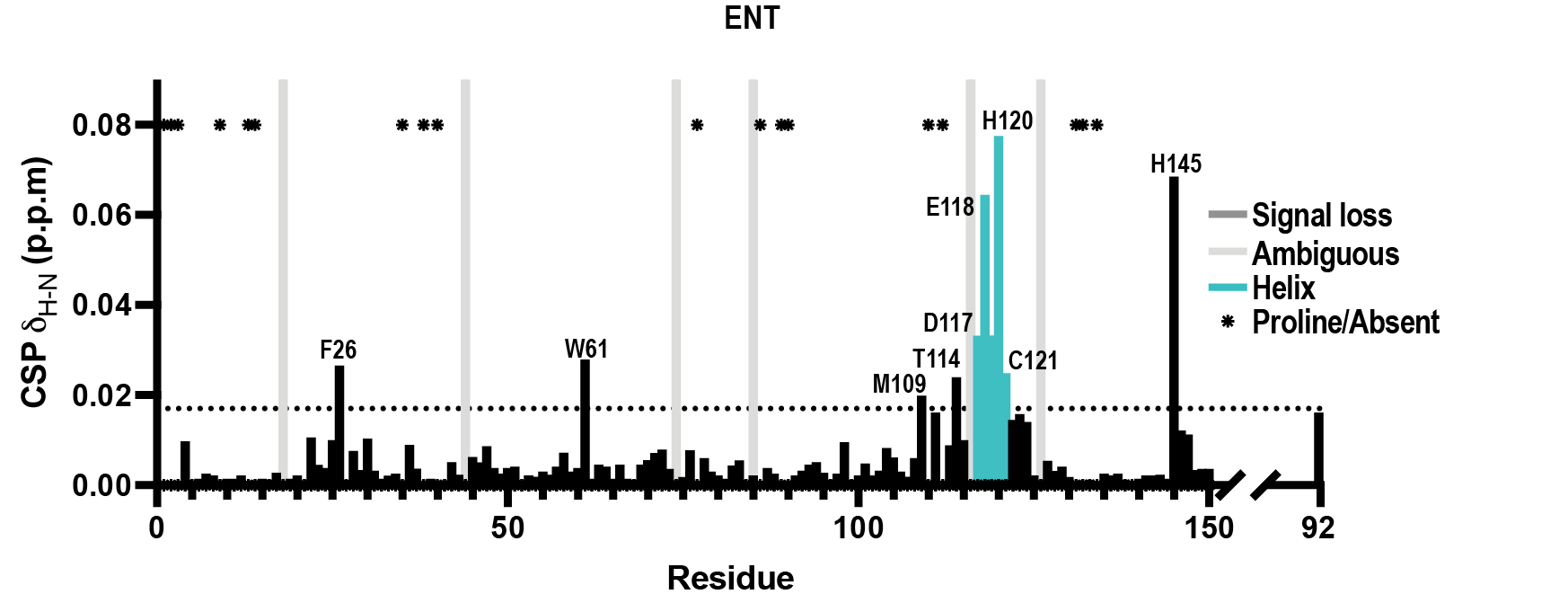
**

**b**

**
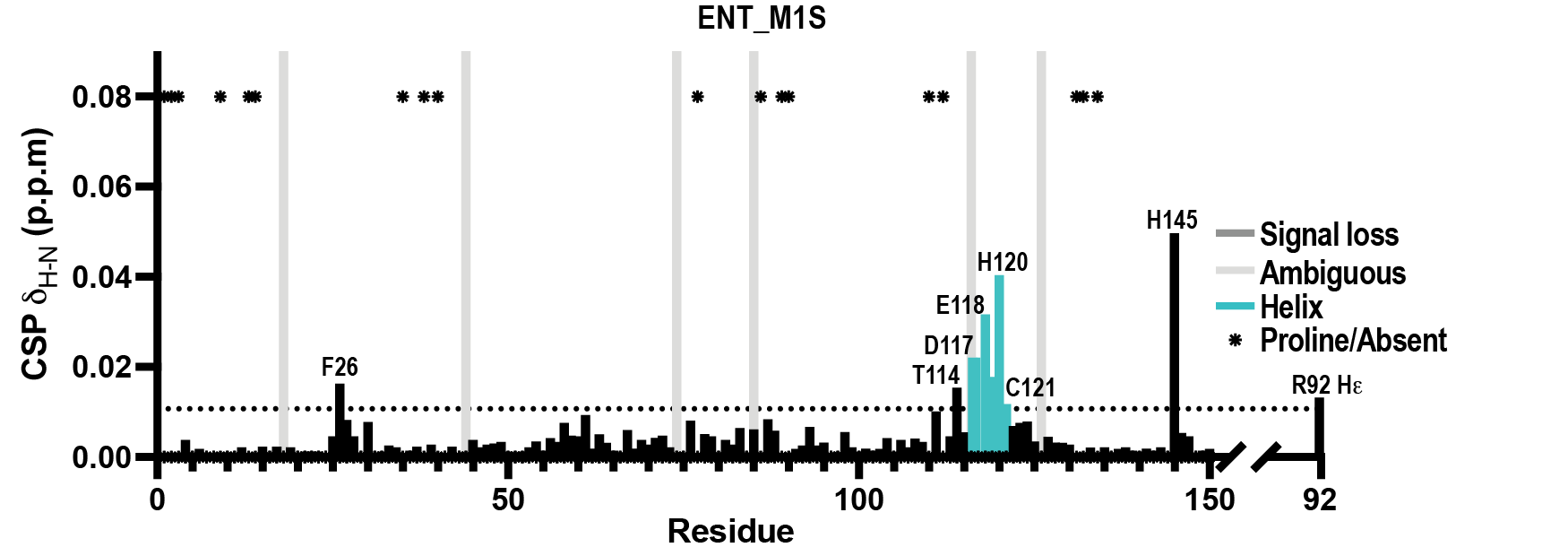
c**

**Supplementary Figure 10: 2D NMR spectra and quantification of hRpn13_ΔMTT binding to ENT or ENT_M1S. a)** ^1^H-^15^N HSQC spectra of 0.1 mM ^15^N-labeled hRpn13_ΔMTT alone (black) and with 20-fold molar excess ENT_M1S (red). Signals that change following peptide addition are labeled and include residues in the helix (blue font), hRpn2-binding surface (purple font) and C-terminal end (orange font). The spectra were acquired at 600 MHz and 25°C in 20 mM sodium phosphate, pH 6.5, 100 mM NaCl, 1 mM TCEP, 10% D_2_O. **b, c**) Changes in chemical shift values for the hRpn13_ΔMTT backbone amide signals are plotted as a function of residue number for b) ENT and c) ENT_M1S. Prolines, M1-S4, and S90, which lacks an assignment, are indicated by asterisks (*). Instances where the chemical shift differences could not be calculated due to ambiguity (light grey bars) or signal loss (dark grey bars) are denoted. A dotted line represents the value at one standard deviation above average. Residues in the Pru helix spanning 117-129 are colored cyan. CSP, chemical shift perturbation; ppm, part per million. **d, e)** CSP data for d) ENT and e) ENT_M1S mapped onto a ribbon representation of hRpn13 Pru bound to ubiquitin (yellow) and hRpn2 (green). Residues with backbone amide signals that shift by greater than one standard deviation above average are displayed by a stick model and labeled. The Pru coloring follows the figure legend with residues for which data are not available indicated in black. PDB 5V1Y was used to generate this figure. Source data for **b** and **c** are provided in the Source Data file.

**
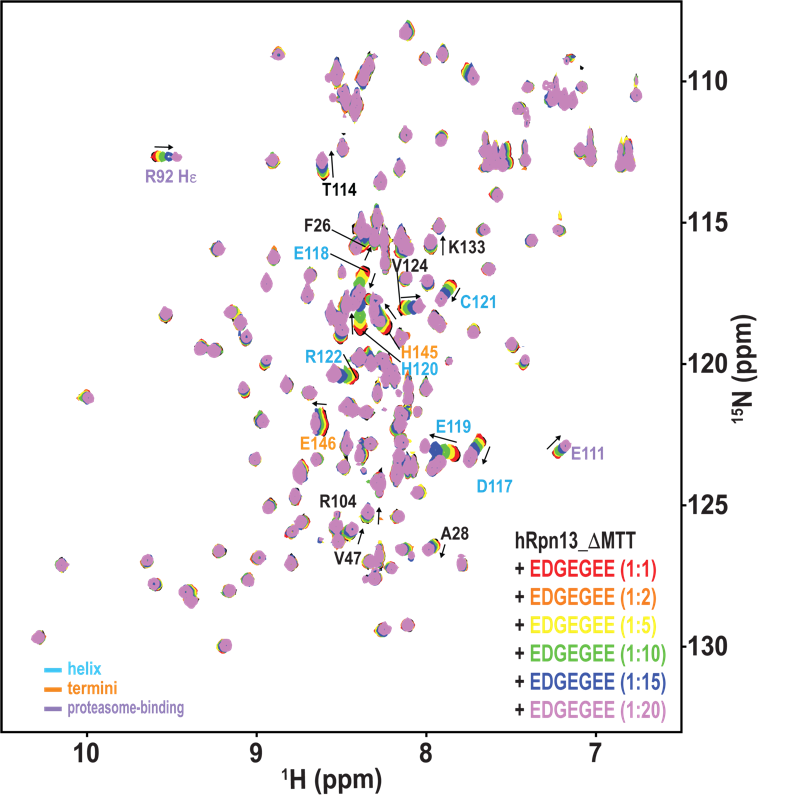

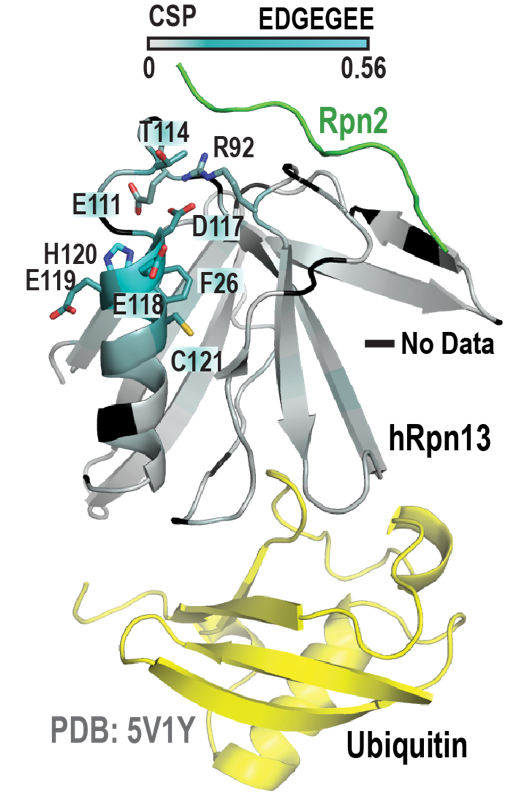
a c**

**
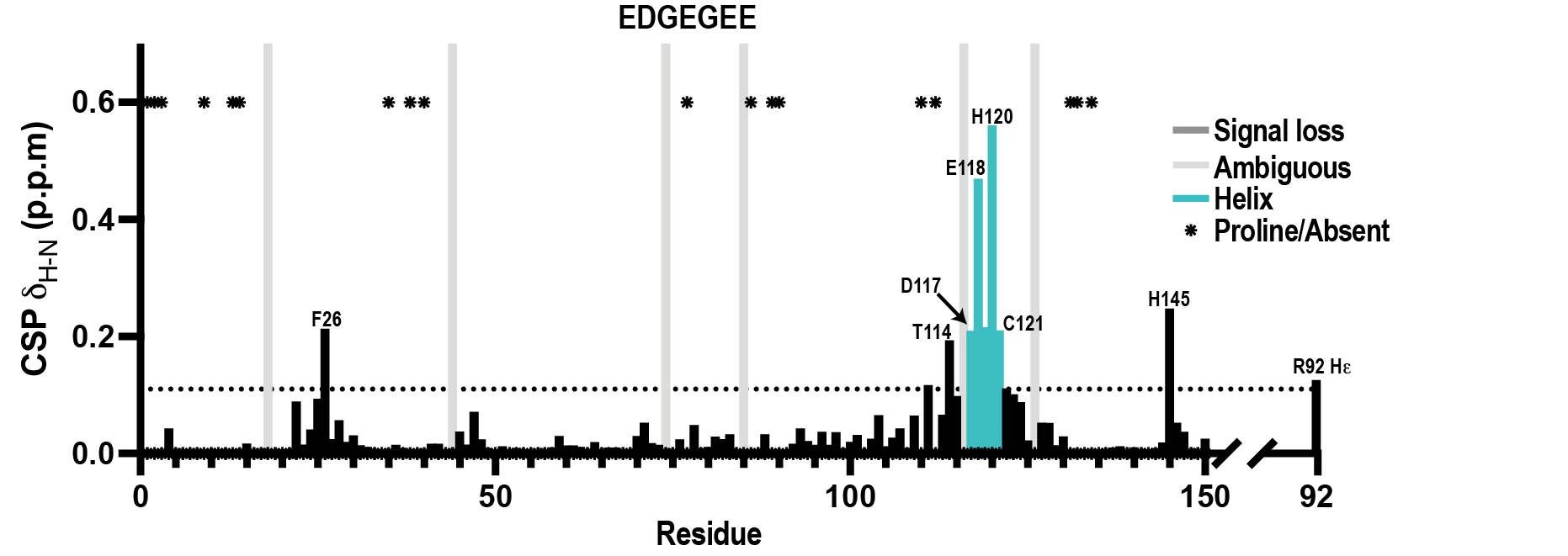
b**

**Supplementary Figure 11: hRpn13 Pru binds to an acidic peptide of sequence EDGEGEE. a)** ^1^H-^15^N HSQC spectra of 0.14 mM ^15^N-labeled hRpn13_ΔMTT alone (black) and with 20-fold molar excess unlabeled EDGEGEE. Select residues are labeled, with those from the helix in blue font. The spectra were acquired on a 600 MHz spectrometer equipped with a cryogenically cooled probe and at 25°C in 20 mM sodium phosphate, pH 6.5, 100 mM NaCl, 1 mM TCEP, 10% D_2_O. **b)** Changes in chemical shift values for backbone amide signals are plotted as a function of residue number for EDGEGEE addition to hRpn13_ΔMTT. Prolines, M1-S4, and S90, which lacks an assignment, are indicated by asterisks (*). Instances where the chemical shift differences could not be calculated due to ambiguity (light grey bars) or signal loss (dark grey bars) are denoted. A dotted line represents the value at one standard deviation above average. Residues in the Pru helix spanning 117-129 are colored cyan. CSP, chemical shift perturbation; ppm, part per million. **c)** Ribbon representation of hRpn13 Pru bound to ubiquitin (yellow) and hRpn2 (green) colored according to the data in panel **b** as indicated by the figure legend. Residues with backbone amide signals that shift by greater than one standard deviation above average are displayed by a stick model and labeled. PDB 5V1Y was used to generate this figure. Source data for **b** is provided in the Source Data file.

**
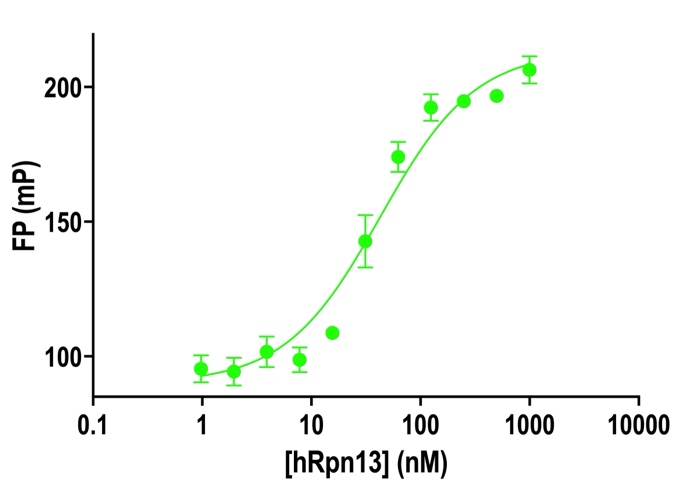
**

**
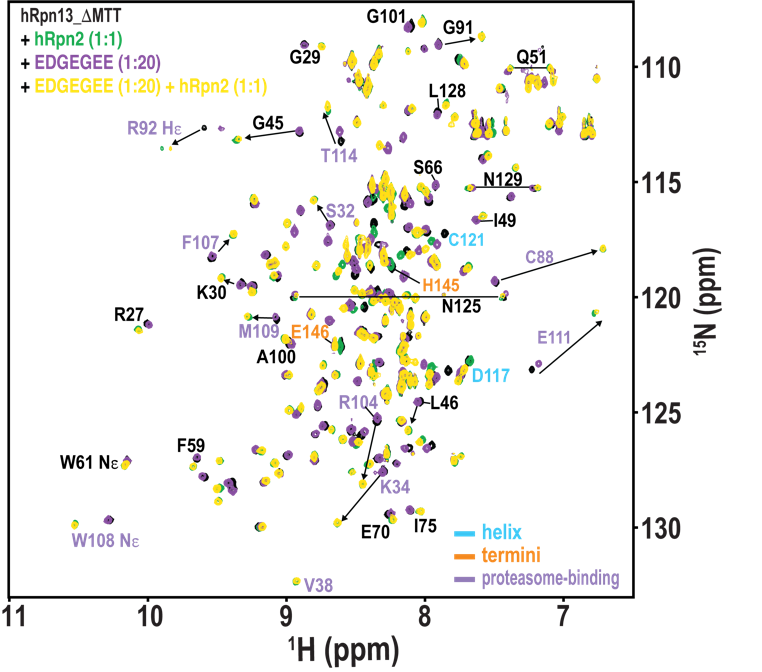
a b**

**
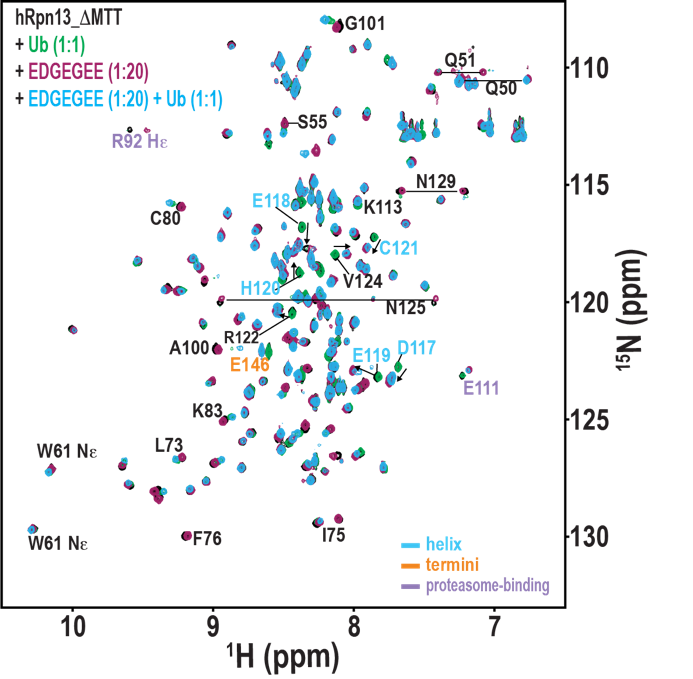
 d**

**
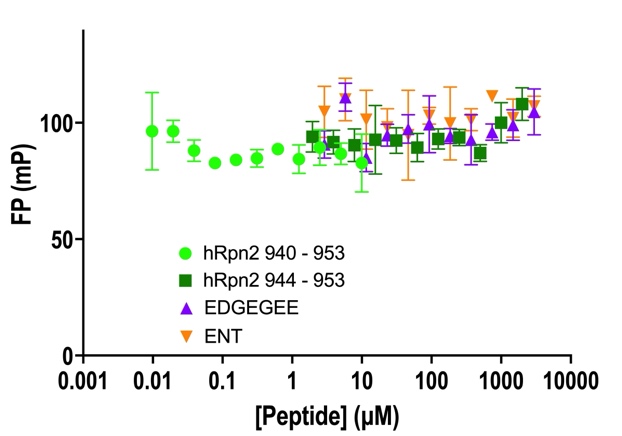
c**

**Supplementary Figure 12: NMR and fluorescence polarization data evaluating hRpn13_ΔMTT binding to hRpn2, ENT, EDGEGEE, and ubiquitin. a)** ^1^H-^15^N HSQC spectrum of 0.1 mM ^15^N-labeled hRpn13_ΔMTT alone (black) or with equimolar hRpn2 (940-953, green), 20-fold molar excess EDGEGEE (purple), or equimolar hRpn2 and 20-fold molar excess EDGEGEE (yellow). Select residues are labeled. **b)** Unlabeled hRpn13_ΔMTT increases the fluorescence polarization of 10 nM TAMRA-labeled hRpn2 (940-953). Points are reported as mean ± SD, N = 3 (assay replicates). **c)** Unlabeled ENT, EDGEGEE, hRpn2 (940-953), and hRpn2 (944-953) do not increase fluorescence polarization of 10 nM TAMRA-labeled hRpn2 (940-953) in the absence of hRpn13. Points are reported as mean ± SD, N = 6 (assay replicates). **d)** ^1^H-^15^N HSQC spectra of 0.1 mM ^15^N-labeled hRpn13_ΔMTT alone (black) or with equimolar ubiquitin (green), 20-fold molar excess EDGEGEE (purple), or both (blue). All spectra were acquired on a 600 MHz spectrometer equipped with a cryogenically cooled probe and at 25°C samples with samples dissolved in 20 mM sodium phosphate, pH 6.5, 100 mM NaCl, 1 mM TCEP, 10% D_2_O. Source data for **b** and **c** are provided in the Source Data file.


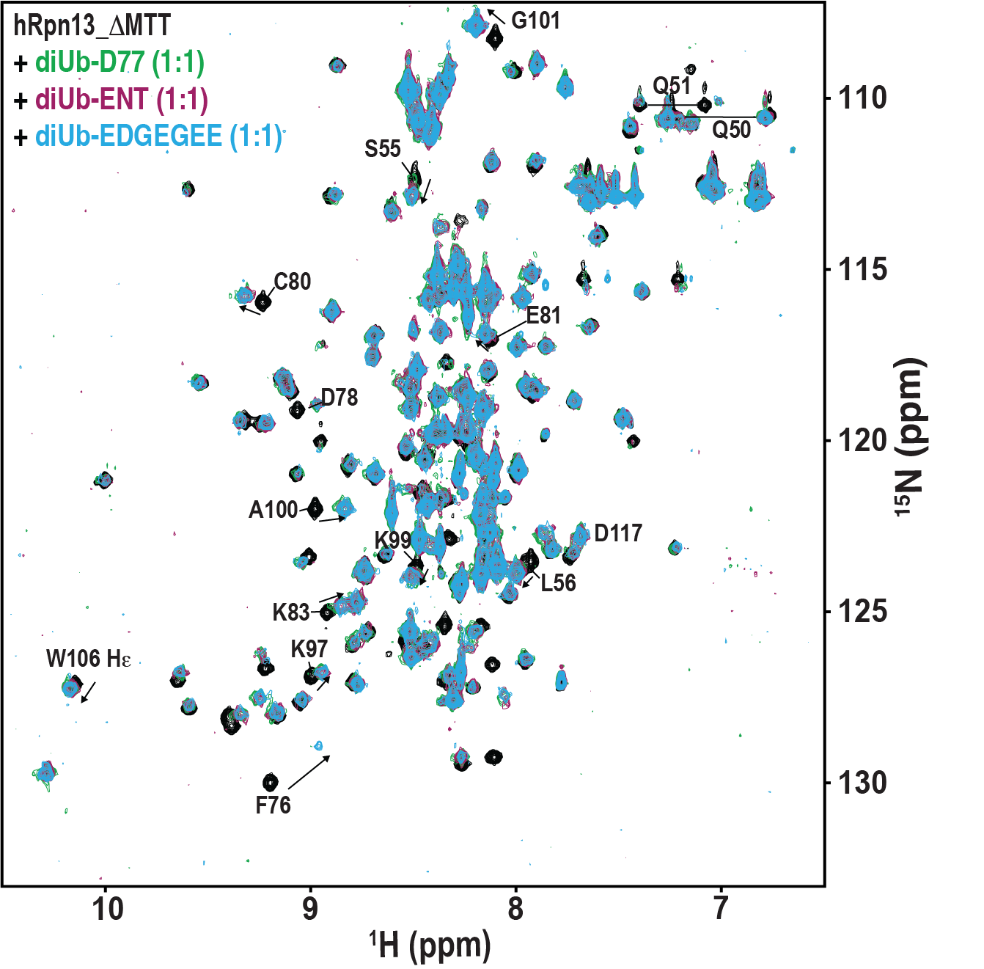


**Supplementary Figure 13: NMR experiments of hRpn13 Pru binding to K48-diUb^EDGEGEE^, K48-diUb^ENT^ or K48-diUb^D77^. a)** ^1^H-^15^N HSQC spectrum of 0.1 mM ^15^N-labeled hRpn13_ΔMTT alone (black) or with equimolar K48-diUb^EDGEGEE^ (blue), K48-diUb^ENT^ (red) or K48-diUb^D77^ (green). Select residues are labeled. The spectra were acquired on a 600 MHz spectrometer equipped with a cryogenically cooled probe and at 25°C samples with samples dissolved in 20 mM sodium phosphate, pH 6.5, 100 mM NaCl, 1 mM TCEP, 10% D_2_O.


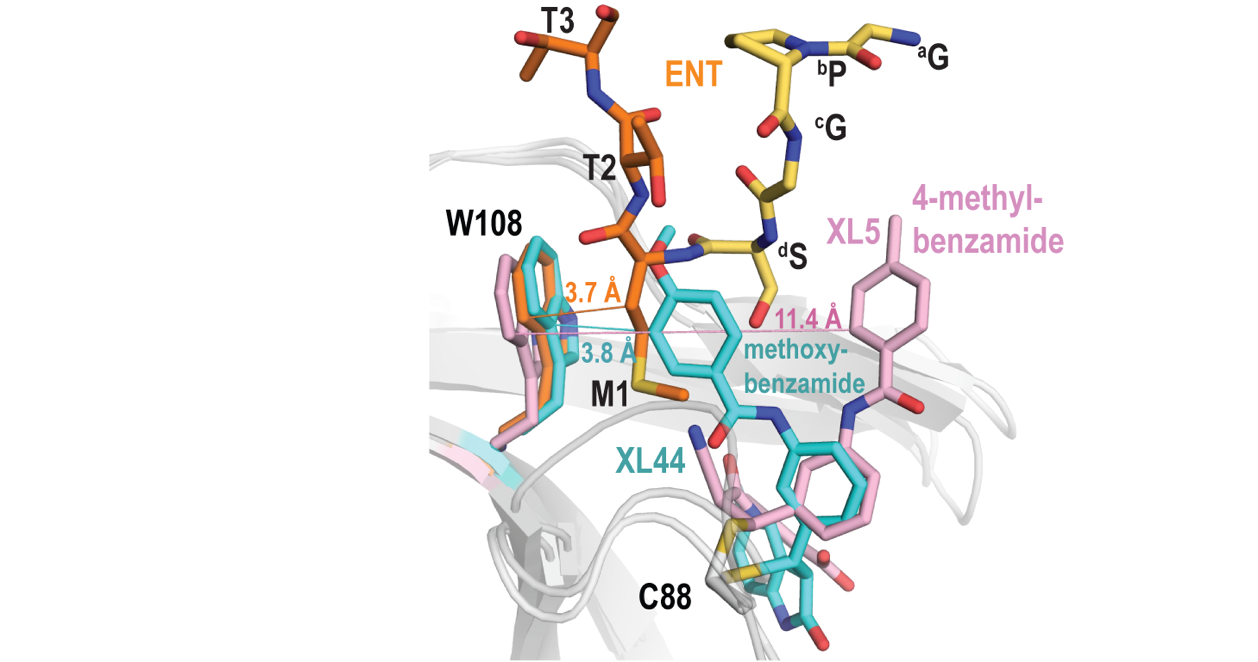


**Supplementary Figure 14: ENT binds Pru at the edge of the XL5 and XL44 binding sites.** Expanded view of overlaid structures of Pru bound to ENT (orange and yellow), **XL5** (pink), or **XL44** (cyan). Distances in Å are displayed between hRpn13 W108 C3ε atom and ENT (M1), **XL5** (4-methyl-benzamide) or **XL44** (methoxy-benzamide). PDB ID 7KXI (**XL5**-bound hRpn13) and 8FTQ (**XL44**-bound hRpn13) were used to generate this figure.

**Supplementary Table 1.** List of atomic distances between hRpn13 Pru-A and ENT within a 4 Å cutoff.

| hRpn13 Pru-A | | | ENT | | |  |
| --- | --- | --- | --- | --- | --- | --- |
| Atom | Residue | Residue no. | Atom | Residue | residue no. | Distance (Å) |
| Cβ | Pro | 40 | O | Ser | d | 3.56 |
|  |  |  | Cγ | Met | 1 | 3.67 |
| Cγ |  |  | Cβ | Ser | d | 3.73 |
|  |  |  | Oγ |  |  | 3.61 |
| Cδ |  |  | Cβ |  |  | 3.9 |
| Cγ | Lys | 42 | O |  |  | 3.76 |
| Cδ |  |  |  | Thr | 2 | 3.76 |
| Cε |  |  |  | Gly | c | 3.17 |
|  |  |  | C | Ser | d | 3.67 |
|  |  |  | O |  |  | 3.22 |
|  |  |  |  | Thr | 2 | 3.41 |
| Nζ |  |  | Cγ | Pro | b | 3.76 |
|  |  |  | Cδ |  |  | 3.82 |
|  |  |  | C | Gly | c | 3.84 |
|  |  |  | O |  |  | 2.66 |
|  |  |  |  | Thr | 2 | 2.74 |
| C | Ser | 90 | Cε | Met | 1 | 3.62 |
| O |  |  | Cβ |  |  | 3.9 |
|  |  |  | Cε |  |  | 3.63 |
| N | Gly | 91 |  |  |  | 3.69 |
| Cα |  |  |  |  |  | 3.77 |
| Cγ2 | Val | 93 | Sδ |  |  | 3.85 |
| Cβ | Trp | 108 |  |  |  | 3.59 |
| Cγ |  |  | Cγ |  |  | 3.88 |
|  |  |  | Sδ |  |  | 3.73 |
| Cδ2 |  |  | Cγ |  |  | 3.65 |
| Cε3 |  |  | Cβ |  |  | 3.8 |
| Cζ3 |  |  | O |  |  | 3.44 |
| CH2 |  |  |  |  |  | 3.78 |
