# Supplementary material for: An adaptive peptide-binding site in ubiquitin receptor hRpn13 revealed by structural studies
Source: Nat Commun. 2025 Jul 1;16:5669. doi: 10.1038/s41467-025-60843-w (PMC12218921; doi:10.1038/s41467-025-60843-w)
Supplement: Supplementary file 2 — Description of Additional Supplementary Files [file 41467_2025_60843_MOESM2_ESM.pdf]

Description of Additional Supplementary Files

File Name: Supplementary Data 1

Description: Trimer.zip. Simulation input files for 100 ns molecular dynamics simulation in Desmond for trimer complex. Files include a configuration parameter file (cfg), initial coordinates (cms), simulation protocol (msj) and final coordinates (pdb).

File Name: Supplementary Data 2

Description: Trimer_Extension_1.zip. Simulation input files for extended 200 ns molecular dynamics simulation in Desmond for trimer complex. Files include a configuration parameter file (cfg), initial coordinates (cms), simulation protocol (msj) and final coordinates (pdb).

File Name: Supplementary Data 3

Description: Trimer_Extension_2.zip. Simulation input files for extended 200 ns molecular dynamics simulation in Desmond for trimer complex. Files include a configuration parameter file (cfg), initial coordinates (cms), simulation protocol (msj) and final coordinates (pdb).

File Name: Supplementary Data 4

Description: Trimer_Extension_3.zip. Simulation input files for extended 200 ns molecular dynamics simulation in Desmond for trimer complex. Files include a configuration parameter file (cfg), initial coordinates (cms), simulation protocol (msj) and final coordinates (pdb).

File Name: Supplementary Data 5

Description: Pru-A.zip. Simulation input files for 100 ns molecular dynamics simulation in Desmond for Pru-A. Files include a configuration parameter file (cfg), initial coordinates (cms), simulation protocol (msj) and final coordinates (pdb).

File Name: Supplementary Data 6

Description: Pru-A_Extension_1.zip. Simulation input files for extended 200 ns molecular dynamics simulation in Desmond for Pru-A. Files include a configuration parameter file (cfg), initial coordinates (cms), simulation protocol (msj) and final coordinates (pdb).

File Name: Supplementary Data 7

Description: Pru-A_Extension_2.zip. Simulation input files for extended 200 ns molecular dynamics simulation in Desmond for Pru-A. Files include a configuration parameter file (cfg), initial coordinates (cms), simulation protocol (msj) and final coordinates (pdb).

File Name: Supplementary Data 8

Description: Pru-A_Extension_3.zip. Simulation input files for extended 200 ns molecular dynamics simulation in Desmond for Pru-A. Files include a configuration parameter file (cfg), initial coordinates (cms), simulation protocol (msj) and final coordinates (pdb).

File Name: Supplementary Data 9

Description: Pru-B.zip. Simulation input files for 100 ns molecular dynamics simulation in Desmond for Pru-B. Files include a configuration parameter file (cfg), initial coordinates (cms), simulation protocol (msj) and final coordinates (pdb).

File Name: Supplementary Data 10

Description: Pru-B_Extension_1.zip. Simulation input files for extended 200 ns molecular dynamics simulation in Desmond for Pru-B. Files include a configuration parameter file (cfg), initial coordinates (cms), simulation protocol (msj) and final coordinates (pdb).

File Name: Supplementary Data 11

Description: Pru-B_Extension_2.zip. Simulation input files for extended 200 ns molecular dynamics simulation in Desmond for Pru-B. Files include a configuration parameter file (cfg), initial coordinates (cms), simulation protocol (msj) and final coordinates (pdb).

File Name: Supplementary Data 12

Description: Pru-B_Extension_3.zip. Simulation input files for extended 200 ns molecular dynamics simulation in Desmond for Pru-B. Files include a configuration parameter file (cfg), initial coordinates (cms), simulation protocol (msj) and final coordinates (pdb).
