# Supplementary material for: An adaptive peptide-binding site in ubiquitin receptor hRpn13 revealed by structural studies
Source: Nat Commun. 2025 Jul 1;16:5669. doi: 10.1038/s41467-025-60843-w (PMC12218921; doi:10.1038/s41467-025-60843-w)
Supplement: Supplementary file 15 — Reporting Summary [file 41467_2025_60843_MOESM15_ESM.pdf]

nature portfolio

Corresponding author(s): Kylie Walters

Last updated by author(s): May 19, 2025

# Reporting Summary

Nature Portfolio wishes to improve the reproducibility of the work that we publish. This form provides structure for consistency and transparency in reporting. For further information on Nature Portfolio policies, see our Editorial Policies and the Editorial Policy Checklist.

## Statistics

For all statistical analyses, confirm that the following items are present in the figure legend, table legend, main text, or Methods section.

| n/a | Confirmed | |
|---|---|---|
| ☐ | ☒ | The exact sample size (*n*) for each experimental group/condition, given as a discrete number and unit of measurement |
| ☒ | ☐ | A statement on whether measurements were taken from distinct samples or whether the same sample was measured repeatedly |
| ☒ | ☐ | The statistical test(s) used AND whether they are one- or two-sided<br>*Only common tests should be described solely by name; describe more complex techniques in the Methods section.* |
| ☒ | ☐ | A description of all covariates tested |
| ☒ | ☐ | A description of any assumptions or corrections, such as tests of normality and adjustment for multiple comparisons |
| ☐ | ☒ | A full description of the statistical parameters including central tendency (e.g. means) or other basic estimates (e.g. regression coefficient) AND variation (e.g. standard deviation) or associated estimates of uncertainty (e.g. confidence intervals) |
| ☒ | ☐ | For null hypothesis testing, the test statistic (e.g. *F*, *t*, *r*) with confidence intervals, effect sizes, degrees of freedom and *P* value noted<br>*Give P values as exact values whenever suitable.* |
| ☒ | ☐ | For Bayesian analysis, information on the choice of priors and Markov chain Monte Carlo settings |
| ☒ | ☐ | For hierarchical and complex designs, identification of the appropriate level for tests and full reporting of outcomes |
| ☒ | ☐ | Estimates of effect sizes (e.g. Cohen's *d*, Pearson's *r*), indicating how they were calculated |

*Our web collection on statistics for biologists contains articles on many of the points above.*

## Software and code

Policy information about availability of computer code

| | |
|---|---|
| Data collection | Schrödinger software, Bruker TopSpin 3.6.5, TECAN Spark microreader (TECAN, Männedorf, Switzerland) , CLARIOstar (BMG LABTECH), Omega 640 spectrophotometer, Southeast Regional Collective Access Team (SER-CAT) using beamline ID-22 (wavelength 1.0 Å) available at the Advanced Photon Source, Argonne National Laboratory equipped with an EIGER 16M detector. |
| Data analysis | Schrödinger software (2022.4), NMRpipe (11.5), NMR-FAM-SPARKY (3.190), NMRbox, PyMOL (3.1.5.1), GraphPad Prism10 (10.3.1), SER-CAT auto-processing software, MolRep, and REFMAC5 (CCP4 package), Coot, Python (3.8.1). |

For manuscripts utilizing custom algorithms or software that are central to the research but not yet described in published literature, software must be made available to editors and reviewers. We strongly encourage code deposition in a community repository (e.g. GitHub). See the Nature Portfolio guidelines for submitting code & software for further information.

## Data

Policy information about availability of data

All manuscripts must include a data availability statement. This statement should provide the following information, where applicable:

- Accession codes, unique identifiers, or web links for publicly available datasets
- A description of any restrictions on data availability
- For clinical datasets or third party data, please ensure that the statement adheres to our policy

The structural coordinates for hRpn13 Pru in complex with ENT have been deposited in the RCSB Protein Data Bank (PDB) under accession code 8VWO. Plasmids for ADRM1_MTT_Del_pGEX-4T-1, pET_3a_Ub_ENT and pET_3a_Ub_EDGEGEE for expression of hRpn13_ΔMTT, Ub-ENT or Ub-EDGEGEE were deposited with Addgene

with IDs 237412, 234413 and 237414, respectively. Simulation input files and final coordinates have been included in as supplementary files. The raw data files for 26 images are included in the source data file. Source data are provided with this paper. Additional data can be obtained from the corresponding author upon request.

## Research involving human participants, their data, or biological material

Policy information about studies with human participants or human data. See also policy information about sex, gender (identity/presentation), and sexual orientation and race, ethnicity and racism.

| | |
|---|---|
| Reporting on sex and gender | N/A |
| Reporting on race, ethnicity, or other socially relevant groupings | N/A |
| Population characteristics | N/A |
| Recruitment | N/A |
| Ethics oversight | N/A |

Note that full information on the approval of the study protocol must also be provided in the manuscript.

# Field-specific reporting

Please select the one below that is the best fit for your research. If you are not sure, read the appropriate sections before making your selection.

[x] Life sciences [ ] Behavioural & social sciences [ ] Ecological, evolutionary & environmental sciences

For a reference copy of the document with all sections, see nature.com/documents/nr-reporting-summary-flat.pdf

# Life sciences study design

All studies must disclose on these points even when the disclosure is negative.

| | |
|---|---|
| Sample size | No statistical methods were used to predetermine sample size. Sample size for each experiment was based on the literature or previous experiences (Lu, X. et al. Structure-guided bifunctional molecules hit a DEUBAD-lacking hRpn13 species upregulated in multiple myeloma. Nat Commun 12, 7318 (2021); Lu, X. et al. Structure of the Rpn13-Rpn2 complex provides insights for Rpn13 and Uch37 as anticancer targets. Nat Commun 8:15540 (2017)) to get statistical significance or reproductivity. |
| Data exclusions | No data were excluded from the analysis |
| Replication | 2D NMR experiments were repeated at least once. Fluorescence polarization experiments were repeated six times. Extended MD simulations were repeated three times. All replications were consistent unless otherwise noted (MD of single Pru:ENT complexes). The structure determination was performed with one crystal. |
| Randomization | This study did not allocate experimental groups thus randomization is not required in the reported experiments. |
| Blinding | Virtual screening, biophysical assays, and structural determination are used in this study. No human, animal or behavioral experiments were performed in this study, thus blinding was not required in this study. All experiments were done with appropriate positive or negative controls as indicated. |

# Reporting for specific materials, systems and methods

We require information from authors about some types of materials, experimental systems and methods used in many studies. Here, indicate whether each material, system or method listed is relevant to your study. If you are not sure if a list item applies to your research, read the appropriate section before selecting a response.

## Materials & experimental systems

| n/a | Involved in the study | |
|---|---|---|
| [x] | [ ] | Antibodies |
| [x] | [ ] | Eukaryotic cell lines |
| [x] | [ ] | Palaeontology and archaeology |
| [x] | [ ] | Animals and other organisms |
| [x] | [ ] | Clinical data |
| [x] | [ ] | Dual use research of concern |
| [x] | [ ] | Plants |

## Methods

| n/a | Involved in the study | |
|---|---|---|
| [x] | [ ] | ChIP-seq |
| [x] | [ ] | Flow cytometry |
| [x] | [ ] | MRI-based neuroimaging |

## Plants

| | |
|---|---|
| Seed stocks | No plants are used in this study |
| Novel plant genotypes | N/A |
| Authentication | N/A |
